# High Levels of PM10 Reduce the Physical Activity of Professional Soccer Players

**DOI:** 10.3390/ijerph20010692

**Published:** 2022-12-30

**Authors:** Michał Zacharko, Robert Cichowicz, Adam Depta, Paweł Chmura, Marek Konefał

**Affiliations:** 1 Department of Human Motor Skills, Wroclaw University of Health and Sport Sciences, I.J. Paderewskiego 35, 51-612 Wrocław, Poland; 2 Institute of Environmental Engineering and Building Installations, Faculty of Civil Engineering, Architekture and Environmental Engineering, Lodz University of Technology, Al. Politechniki 6, 90-924 Lodz, Poland; 3 Department of Forecasts and Quantitative Analyses, Faculty of Organization and Management, Institute of Management, Lodz University of Technology, Wolczanska Street 221, 93-005 Lodz, Poland; 4 Department of Medical Insurance and Health Care Financing, Medical University of Lodz, Lindleya 6, 90-131 Lodz, Poland; 5 Department of Team Games, Wroclaw University of Health and Sport Sciences, I.J. Paderewskiego 35, 51-612 Wrocław, Poland

**Keywords:** football, total distances covered, high speed running, intensity, air quality, particulate matter, regions

## Abstract

The aim of this study is to determine the impact of air quality, analyzed on the basis of the PM10 parameter in three regions of Poland, on the physical activity of soccer players from the Polish Ekstraklasa. The study material consisted of 4294 individual match observations of 362 players during the 2019/2020 domestic season. The measured indices included the parameter of air quality—PM10—and players’ physical activities: total distance (TD) and high-speed running (HSR). Poland was divided into three regions (North, Central, South). The statistical analysis of particulate matter (PM) and athletes’ physical activities, compared by region, revealed the effects in relation to the PM10 (H = 215.6566(2); *p* = 0.0001) and TD (H = 28.2682(2); *p* = 0.0001). Players performed better in regards to physical parameters in the North Region, where air pollution is significantly lower than in other regions. This means that even a short stay in more polluted regions can reduce the performance of professional footballers, which can indirectly affect the outcome of the match. Therefore, greater actions should be taken to improve air quality, especially through changes in daily physical activity, as this will reduce the carbon footprint.

## 1. Introduction

Air pollution is a factor that is currently attracting greater attention because of its threat to human health (Schraufnagel et al., 2019) [1]. According to the Lancet Commission on Pollution and Health, pollution is currently the principal environmental cause of disease and premature death in the world. Pollution-induced diseases were responsible for around 9 million premature deaths in 2015 (Landrigan et al., 2018) [2] and 790 000 additional deaths in Europe alone (Lelieveld et al., 2019) [3]. Moreover, air pollution is the most important risk factor among all environmental pollutants (Cohen et al., 2017) [4]. One of the most harmful parameters is particulate matter (PM), which is produced by burning wood and fossil fuels, especially due to construction work and traffic (Cichowicz and Stelęgowski, 2019) [5]. Its concentration depends on several factors, including the season of the year, time of day, and location (Nieckarz and Zoladz, 2020) [6]. It is worth noting that in large urban agglomerations in many cities around the world, increasingly higher concentrations of PM are observed (Gupta et al., 2006; Khilnani and Tiwari, 2018; Tian and Sun, 2017) [7,8,9]. In Poland, the concentration of PM and other parameters of air pollutants varies depending on the region (Lubiński et al., 2005) [10]. Breathing air with a high concentration of pollutants has a negative effect on health (Orru et al., 2017) [11]. This effect has been extensively studied, and subsequent studies have consistently documented the negative effects of pollution on people’s physical and mental health (Landrigan et al., 2018; Welsch, 2007) [2,12]. Particulate matter is especially dangerous because it recruits immune cells, increases oxidative stress in both the vascular system and the brain, and makes the vascular system hypersensitive to vasoconstrictors, contributing to vascular (endothelial) dysfunction (Münzel et al., 2018) [13]. It is also worth emphasising that air pollution has a negative impact on several components of an individual’s mental health, including subjective well-being (Li et al., 2018) [14], life satisfaction (Welsch, 2006) [15], happiness (Welsch, 2007) [12], and even depressive symptoms (Zhang et al., 2017) [16]. These harmful effects of air pollution also apply to sports and physical activity, which is why attention should be paid to examining the impact that pollution can have on an individual’s health and level of physical activity (Roberts et al., 2014) [17].

Previous research has shown that there are many other factors affect an individual’s physical (Marmot, 2005) [18] and mental health (Dolan et al., 2008) [19]. An example of such a factor is physical activity, which has a positive impact on both physical (Downward et al., 2016; Humphreys et al., 2014) [20,21] and mental health (Downward and Dawson, 2016; Wicker and Frick, 2017) [22,23]. For this reason, a topic that is currently receiving special attention is the impact of air pollution on the health of people who engage in physical activity (An et al., 2018; Giles and Koehle, 2014) [24,25] and practice outdoor sports, including athletes (Kuskowska et al., 2019; Reche et al., 2020) [26,27]. However, it should be emphasized that all forms of physical activity increase the amount of air ventilated through the lungs (minute ventilation—VE), which is several times greater during moderate-intensity exercise than at rest (Bowen et al., 2019; Zoladz et al., 2019) [28,29]. For example, minute ventilation (VE), which is about 6–8 L of air for a person at rest, can reach 30–50 L per minute during moderate exertion and may even exceed 100 L per minute during very intense exercise (Wasserman et al., 2011) [30]. Some athletes are able to exceed the VE value by up to 200 L per minute, which is about 30 times more than at rest levels (Allen, 2004) [31]. During increased intake of air, the amount of suspended solid particles inhaled is greater. This, in turn, results in the deposition of more these substances in the respiratory tract and other body organs (Nieckarz and Zoladz, 2020) [6].

Therefore, the study of soccer players is indicated, because they are particularly exposed to the negative health effects of air pollution. The number and frequency of professional soccer matches is large. Match schedules are very exhausting and teams need to be ready to play up to 60 matches per season (Carling et al., 2018) [32]. During a 90 min game, elite-level players run approximately 10 km and perform numerous explosive bursts of activities, such as kicking, jumping, tackling, sprinting, changing direction, turning, and sustaining forceful contractions to maintain balance and control of the ball against defensive pressure (Stølen et al., 2005) [33]. Therefore, every match requires players to be in top physical condition. The parameters of physical activity most frequently studied and described in the literature are total distance covered (TD) and high-speed running (HSR) (Aquino et al., 2021; Konefał et al., 2021) [34,35]. For example, Andrzejewski et al., (2018) [36] proved that total distance covered is significantly greater for winning teams. In other studies, both Chmura et al., (2018) [37] and Modric et al., (2019) [38] indicated that high-intensity efforts (sprinting and fast running) should be included among the most important measures of physical activity in soccer. The aim of this study is to determine the impact of air quality, analyzed on the basis of the PM10 parameter in three regions of Poland, on the physical activity of soccer players from the Polish Ekstraklasa.

## 2. Materials and Methods

### 2.1. Match Sample and Data Collection

Match performance data were collected from 362 soccer players competing in the Polish Ekstraklasa during the 2019/2020 season. The league featured 16 teams, who faced each opponent twice during each season, at home and away. Additionally, after playing 30 matches, two groups were formed: the championship and the relegation group, and in each of these, 7 additional matches were played. Thus, a season included 37 match days and 296 matches. A total of 4294 individual match observations of outfield players (excluding goalkeepers, due to the specificity of the position) were made (Konefał et al., 2019) [39]. Only data on players who completed entire matches (i.e., remained on the pitch for the entire 90 min) were taken into account.

The physical activity data were collected using the previously-validated (Linke et al., 2020) [40] TRACAB system (ChyronHego, NY, USA). This system consists of two multi-camera units, each consisting of three HD-SDI cameras with a resolution of 1920 × 1080 pixels. The sampling frequency of this system was 25 Hz. Two variables were analyzed: total distance (TD), distance covered in high-speed running (HSR; 19.8–25.1 km·h^−1^). The TRACAB tracking system has been verified by passing the official FIFA (Fédération Internationale de Football Association) test protocol for electronic performance and tracking systems (EPTS).

This study maintains the anonymity of the players (following data protection laws), is conducted in compliance with the Declaration of Helsinki, and was approved by the Senate Committee on Ethics of Scientific Research at the Academy of Physical Education in Wroclaw (No. 12/2021).

### 2.2. Procedures

Data on air quality were collected on the basis of information from automatic air monitoring stations, which were made available by the Voivodship Inspectorate for Environmental Protection (WIOŚ) and by the Main Inspectorate for Environmental Protection (GIOŚ) in Poland, whereas the meteorological data used in this analysis come from the Institute of Meteorology and Water Management-National Research Institute. The parameter PM10 was analyzed because its concentration is one of the basic parameters examined in the assessment of air quality (Anderson et al., 2012; Zaric et al., 2021) [41,42]. However, according to the European Union (Directive 2008/50/EC), the permissible annual average concentration of PM10 is 40 µg·m^−3^, and the daily average concentration is 50 µg·m^−3^. However, according to WHO (WHO, 2021) [43], the permissible annual average concentration of PM10 is 15 µg·m^−3^, and the daily average is 45 µg·m^−3^.

Data were collected from air quality measurement stations located closest to the stadiums where matches were played, and all measurements were read with an accuracy of 0.01 µg·m^−3^. In each analyzed match, three measurements of air pollution were made (at the beginning, during the break, and at the end of the match). The arithmetic mean and standard deviation were then calculated from these air pollution values.

**Figure 1:**
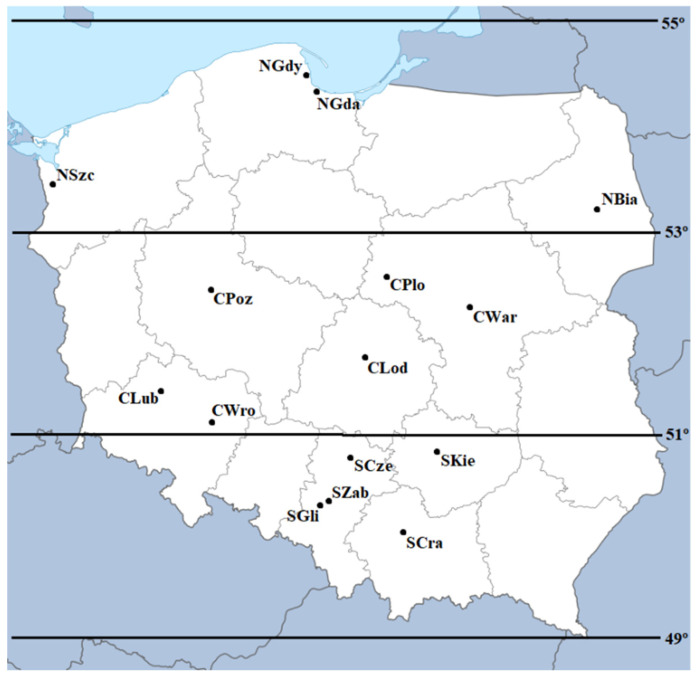
Location of selected measuring stations in selected cities of Poland.

Data on the analyzed pollutants came from a total of 15 monitoring stations, which were divided into three regions of Poland (North Region, Central Region, South Region). As a result, regions with different levels of air pollution were obtained (Lubiński et al., 2005) [10]. Regions have been designated based on latitudes (Cox and Popken, 2020) [44], with each region extending 2° north latitude (N). Regarding the North Region (latitude 53° N–55° N), the players play matches in the cities of Bialystok, Gdansk, Gdynia, and Szczecin (1038 observations). In the Central Region (latitude 51° N–53° N), teams play matches in the cities of Lodz, Lubin, Plock, Poznan, Wroclaw, and Warsaw (1624 observations). In the South Region (latitude 49° N–51° N), the teams perform in the cities of Cracow, Czestochowa, Gliwice, Kielce, and Zabrze (1632 observations). Thus, the locations were obtained and marked with a combination of symbols relating to the region and the city (Table 1 and Figure 1).

### 2.3. Statistical Analyses

In the research, several methods of statistical inference were used, including the Shapiro–Wilk normality test, tests of homogeneity of variance, i.e., Bartlett’s, Cochran’s, and Hartley’s tests (used to verify the assumptions: about the normality of the explained variable distribution and the homogeneity of its variance in the studied populations), and the Kruskal–Wallis test, in the case of the data failing to meet the above assumptions.

In order to apply the analysis of variance for the variables—HSR, TD, PM10—initially, the assumption regarding the normality of the distribution of the above-mentioned variables was checked using the Shapiro–Wilk test. In order to verify the null hypothesis regarding the distribution normality of the results of the analyzed variables, the null hypothesis that the examined feature of the population has a normal distribution was checked against the alternative hypothesis that the feature of the population does not have a normal distribution. At the significance level of α = 0.05, the verified null hypothesis was rejected, so it could not be concluded that the distribution of the variables was normal. In the next stage, the assumption regarding the homogeneity of the variance of variables in the regions was checked. At the significance level of α = 0.05, the verified null hypothesis that the variances in individual regions are the same for the analyzed variables was rejected. Due to the failure of the above assumptions regarding the classical analysis of variance, the non-parametric Kruskal–Wallis test was used.

All statistical analyses were performed using the Statistica ver. 13.3 software package (Dell Inc., Tulsa, OK, USA).

## 3. Results

Based on the results presented in Table 2, it can be concluded that the variables TD and PM10 depend on the regions (*p* < 0.05).

The statistical analysis of PM and the physical activity of players, compared by regions (North, Central, South), revealed the effects in relation to the PM10 (H = 215.6566(2); *p* = 0.0001), TD (H = 28.2682(2); *p* = 0.0001). No significant effect was found for HSR (H = 3.411(2); *p* = 0.1817); Table 2.

## 4. Discussion

The aim of the study is to determine the impact of air quality, analyzed on the basis of the PM10 parameter in three regions of Poland, on the physical activity of soccer players from the Polish Ekstraklasa. On the basis of the literature reviewed, the authors noted that only two studies have been published on the impact of air quality on the activity of professional soccer players (Lichter et al., 2017; Zacharko et al., 2021) [45,46]. This study is a continuation of an important observation described in the publication entitled, “Air Pollutants Reduce the Physical Activity of Professional Soccer Players” (Zacharko et al., 2021) [46]. Continuing the research in this area is very important, as it concerns the physical activity of professional athletes, but it can also support the health-promoting nature of the daily physical activity of the whole society. The quantitative and qualitative analysis of soccer performance is currently very popular, as it can maximize the chances for team success (Maneiro et al., 2020) [47].

Lichter et al. (2017) [45] proved that air pollution negatively affects footballers in German stadiums. However, the performance indicator was limited to only the number of passes attempted during the match. As soccer is considered a high-intensity sport, the total distance covered and the running speed are more valuable parameters for assessing the performance of the athletes in order to evaluate the impact of air quality (Barnes et al., 2014) [48]. Therefore, in another study, based on the example of the German Bundesliga, the topic of physical activity was discussed, and it was proved that the reduction in the level of air quality during the match had a negative impact not only on technical activities, such as passing, but also on total distance covered (TD) and high-speed running (HSR) (Zacharko et al., 2021) [46]. In order to investigate the problem in more detail, research was carried out in Poland, i.e., a country with more polluted air and characterized by large regional differences in ambient air pollution (Kocot and Zejda, 2022) [49].

Analyzing the average level of particulate matter in three regions of Poland, our study found that the more northern the region, the lower the level of PM10 pollution. The difference may be due to the fact that the South Region is the most industrial region in Poland, which includes the Silesian Agglomeration and Cracow, which in turn are of the cities with the worst air quality in Europe (Traczyk and Gruszecka-Kosowska, 2020) [50]. On the other hand, in the Central Region, there are large cities such as Warsaw, a city more polluted than, for example, Bialystok or Gdansk, which are included in the North Region (Slama et al., 2020) [51]. This is also due to the terrain and its roughness, as well as meteorological conditions. Additionally, attention should be paid to the fact that in Poland, the dominant wind directions are western and south-western, which may result in both the transboundary and local displacement of pollutants, and the consequence of this may be increased levels of pollution in a given area. Under the best air quality conditions (the lowest levels of PM10), i.e., in the North Region of Poland, the players exhibited a significantly longer average TD compared to those noted in the Central Region and South Region. In addition, when analyzing the HSR, it was noticed that in the North Region, players also achieved the best results, although this is not supported by statistical significance. Thus, it can be seen that by playing matches in a less polluted environment, soccer players can achieve better results regarding the physical parameters. This may be caused mainly by geographic and meteorological conditions, as a consequence of lower population density, higher average wind speed, and more green areas. In addition, it is also worth analyzing the human body’s response to breathing polluted air and its impact on the exercise capacity of the players.

All forms of physical activity increase the amount of air ventilated through the lungs (minute ventilation—VE), which is several times higher than during rest, even during moderate-intensity exercise (Allen, 2004; Bowen et al., 2019; Zoladz et al., 2019) [28,29,31]. Additionally, this causes a greater intake of particulate matter into the lungs and increases the amount of particulate matter deposited in the respiratory tract. That is why air quality is especially important during a soccer match because the effect of increased physical exertion causes the absorption of harmful substances from the air into the body (Duda et al., 2020) [52]. Moreover, Kargarfard et al. (2015) [53] showed that hematological parameters and cardiovascular functions during exercise are disturbed by high concentrations of air pollution. In athletes, the consequence is worsening lung function, which in turn results in reduced peak expiratory flow and increased airway inflammation (Qin et al., 2019) [54]. Moreover, elevated blood pressure caused by air pollution can even impaired exercise capacity and decrease athletic performance (An et al., 2018; Tainio et al., 2021) [24,55].

Considering the negative impact of air pollution on the human body, it is worth determining specific actions that should be taken so that athletes and fans are less exposed to the harmful health effects related to poor air quality. Several very interesting concepts were presented by Nieuwenhuijsen (2021) [56], the main suggestion of which was to increase active transport (walking and cycling). By walking or cycling, the carbon footprint produced by daily trips is reduced by up to 84%, compared to that created by car users (Brand et al., 2021) [57]. At the same time, active transport will lead to an increase in physical activity and, as a result, the promotion and improvement of health. This method of movement may be supported by the concept of the so-called 15 min city, in which schools, work, sports, shopping, and entertainment are all within a 15 min walking or cycling distance from home (Moreno et al., 2021) [58]. Another solution could be a car-free city that relies heavily on public, pedestrian, or bicycle transport (Nieuwenhuijsen and Khreis, 2016) [59]. By applying the above concepts, the society (fans) can contribute to the improvement of air quality in cities, and at the same time, affect the performance of players during matches.

The authors are fully aware of numerous factors that could have influenced the results of the presented analyses. The measuring stations were located close to the stadiums; however, to obtain more accurate measurements, the meters should be placed in the stadiums themselves. In addition, as the meteorological conditions and the type of development between the stadium and the measuring station were not taken into account, the air quality data may have been inaccurate. Another limitation is the failure to take into account other parameters that make it possible to characterize the external load, such as acceleration, deceleration, and player load, which also are used to express the demands of matches in non-cyclical team sports. Additionally, the HSR parameter was not individualised based on the percentage of maximum sprint speed, and the match results were not taken into account. In addition, the time spent by the players before the match in a given zone, as well as the diversity of the schedule of the games, were not taken into account. The above limitations are worth considering in future research. Moreover, in subsequent studies, the dynamics of regeneration processes in various air quality should be considered.

## 5. Conclusions

Air pollution is an important situational factor during soccer matches. Even a short-term stay in a more polluted region can reduce the performance of professional soccer players, which can indirectly affect the match outcome. Moreover, it seems that every fan can take action in everyday life to improve air quality. Supporting one’s favorite players and soccer teams should not be limited only to activity in the stadium, but should also extend to daily physical activity, which will reduce the carbon footprint. As a result, this change in daily activity will improve air quality, which will translate into significant health benefits for both athletes and fans.

## Figures and Tables

**Table 1:** Measuring station symbols.

Symbol	Region	City Name	Population
NBia	North	Bialystok	293,413
NGda	North	Gdansk	486,271
NGdy	North	Gdynia	244,676
NSzc	North	Szczecin	394,482
CLod	Central	Lodz	664,860
CLub	Central	Lubin	69,267
CPlo	Central	Plock	113,660
CPoz	Central	Poznan	545,073
CWro	Central	Wroclaw	674,312
CWar	Central	Warsaw	1,863,056
SCra	South	Cracow	802,583
SCze	South	Czestochowa	210,773
SGli	South	Gliwice	172,628
SKie	South	Kielce	184,520
SZab	South	Zabrze	156,935

Population status as of 31 December 2021. Source: Central Statistical Office.

**Table 2:** Value of air pollution and physical activity parameters by region (mean ± SD).

Parameter	Region			SSD *p* < 0.05
	North (N)	Central (C)	South (S)	
PM10 [µg·m^−3^]	18.16 ± 11.70	22.20 ± 12.62	27.33 ± 20.32	N-C; N-S; C-S
Total Distance [km]	10.78 ± 0.83	10.61 ± 0.87	10.59 ± 0.90	N-C; N-S
High Speed Running [m]	669.84 ± 204.55	656.10 ± 214.83	661.43 ± 214.26	-

SSD—statistically significant differences.

## Data Availability

The data used for this study was acquired from a third-party, https://tracabportal.azurewebsites.net/login (access on 1 April 2021). The data was provided under scientific cooperation with a football clubs currently appearing in Ekstraklasa. The authors’ ethical approval also prevents them from sharing any data in any way that could be re-identified. The metadata would allow someone else to re-identify teams and possibly players. However, access to the data should be possible from the third-party. The data acquired were so called ‘excel dumps’ of player statistics per match. Access to the data can be organized by contacting Match Analysis Hub: info@chyronhego.com.
